# Stereotactic body radiotherapy for kidney cancer: a 10-year experience from a single institute

**DOI:** 10.1093/jrr/rrab031

**Published:** 2021-04-19

**Authors:** Takaya Yamamoto, Yoshihide Kawasaki, Rei Umezawa, Noriyuki Kadoya, Haruo Matsushita, Kazuya Takeda, Yojiro Ishikawa, Noriyoshi Takahashi, Yu Suzuki, Ken Takeda, Kousei Kawabata, Akihiro Ito, Keiichi Jingu

**Affiliations:** Department of Radiation Oncology, Tohoku University Graduate School of Medicine, Sendai 980-8574, Japan; Department of Urology, Tohoku University Graduate School of Medicine, Sendai 980-8574, Japan; Department of Radiation Oncology, Tohoku University Graduate School of Medicine, Sendai 980-8574, Japan; Department of Radiation Oncology, Tohoku University Graduate School of Medicine, Sendai 980-8574, Japan; Department of Radiation Oncology, Tohoku University Graduate School of Medicine, Sendai 980-8574, Japan; Department of Radiation Oncology, Tohoku University Graduate School of Medicine, Sendai 980-8574, Japan; Department of Radiation Oncology, Tohoku University Graduate School of Medicine, Sendai 980-8574, Japan; Department of Radiation Oncology, Tohoku University Graduate School of Medicine, Sendai 980-8574, Japan; Department of Radiation Oncology, Tohoku University Graduate School of Medicine, Sendai 980-8574, Japan; Department of Radiation Oncology, Tohoku University Graduate School of Medicine, Sendai 980-8574, Japan; Department of Radiation Oncology, Tohoku University Graduate School of Medicine, Sendai 980-8574, Japan; Department of Urology, Tohoku University Graduate School of Medicine, Sendai 980-8574, Japan; Department of Radiation Oncology, Tohoku University Graduate School of Medicine, Sendai 980-8574, Japan

**Keywords:** stereotactic body radiotherapy (SBRT), kidney cancer, renal cell carcinoma, local control (LC)

## Abstract

The purpose of this retrospective study was to investigate survival outcomes and irradiated tumor control (local control [LC]) and locoregional control (LRC) after stereotactic body radiotherapy (SBRT) for T1 or recurrent T1 (rT1) kidney cancer. Twenty-nine nonconsecutive patients with 30 tumors were included. SBRT doses of 70 Gy, 60 Gy or 50 Gy in 10 fractions were prescribed with a linear accelerator using daily image guidance. The Kaplan–Meier method was used to estimate time-to-event outcomes, and the log-rank test was used to compare survival curves between groups divided by each possible factor. The median follow-up periods for all patients and survivors were 57 months and 69.6 months, respectively. The five-year LC rate, LRC rate, progression-free survival (PFS) rate, disease-specific survival (DSS) rate and overall survival (OS) rate were 94%, 88%, 50%, 96% and 68%, respectively. No significant factor was related to OS and PFS. Three of 24 non-hemodialysis (HD) patients had new-onset-HD because of the progression of underlying kidney disease. Grade 3 or higher toxicities from SBRT did not occur. In conclusion, SBRT for kidney cancer provided a high rate of LC, LRC and DSS with minimal toxicities, but patient selection and indication for SBRT should be done carefully considering the relatively low OS rate.

## INTRODUCTION

The incidence of small tumors of the kidneys has increased for decades with the progression and widespread use of cross-sectional imaging, such as computed tomography (CT), ultrasonography and magnetic resonance imaging (MRI), particularly in elderly patients [1,2]. Surgical resection is the standard treatment for early-stage kidney cancer, but difficulties in performing surgery on elderly patients sometimes occur because of their renal function, comorbidities, past-surgical history, frailty and other factors [3]. Radical nephrectomy and partial nephrectomy, as well as ablative techniques such as cryosurgery and radiofrequency ablation, are curative therapeutic options for stage I kidney cancer. Stereotactic body radiotherapy (SBRT), which is a powerful local treatment technique with minimal invasiveness, is one of the treatment options for oligometastatic lesions in patients with kidney cancer because its effectiveness and safety have been confirmed [3–5]. SBRT for primary kidney cancer has also been reported, but unfortunately, SBRT has not been recommended as a treatment option for stage I kidney cancer in the National Comprehensive Cancer Network (NCCN) guidelines [6]. Furthermore, patients who were treated with SBRT for stage I kidney cancer showed lower survival rates than those treated with partial nephrectomy or ablative techniques [7]. However, additional treatment options would be beneficial for patients. Compared with ablative techniques, SBRT represents a safe treatment, regardless of the location of blood vessels and provides a cure for larger tumors [8]. Because the need for SBRT in patients with kidney cancer is increasing in Japan, which has a rapidly aging society, SBRT for kidney cancer has been approved since 2018 as a treatment covered by the national health insurance program that covers all citizens in Japan. Before insurance coverage, a previous phase 1/2 study of SBRT for kidney cancer was performed in Japan, and our institute joined in 2010 [9]. The result of the previous phase 1/2 study have not been published, therefore, our relatively long-term experience can be useful, especially for Japanese patients because patients who received SBRT for kidney cancer at our institute have been treated using the same protocol regardless of enrollment in the trial. In this study, these patients were retrospectively analyzed. The primary endpoint of this study was to estimate the overall survival (OS) rate, and the secondary endpoints were irradiated tumor control (i.e. local control [LC]), locoregional control (LRC), progression-free survival (PFS), disease-specific survival (DSS) and toxicity.

## MATERIAL AND METHODS

### Data identification and eligibility criteria

**Fig. 1.**
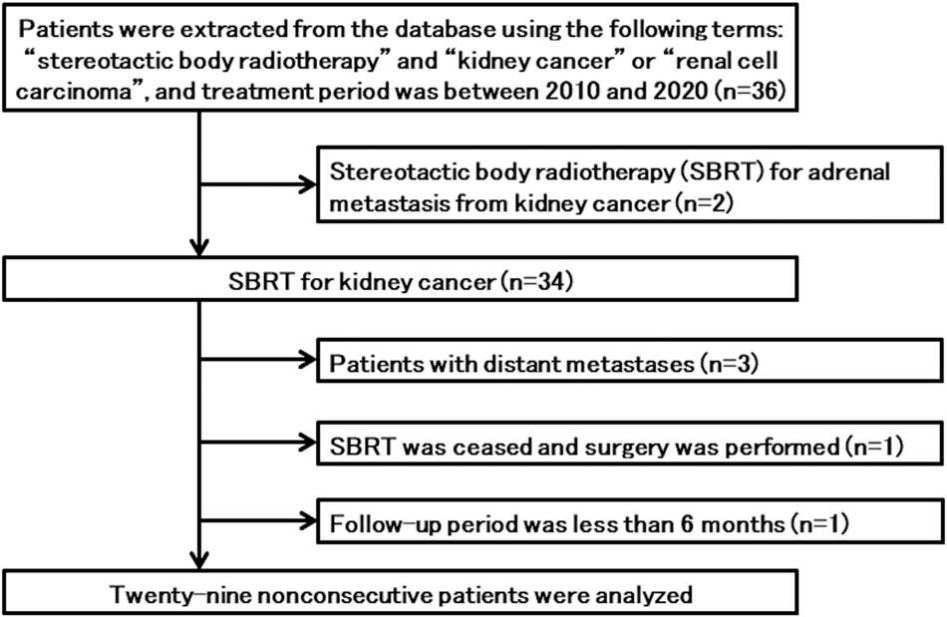
Flow chart of patient selection from the database. One patient ceased SBRT and received radical nephrectomy after hemodialysis was introduced.

Data were searched from our clinical database using the terms ‘stereotactic body radiotherapy’ and ‘Jin-gan’ or ‘Jinsaibo-gan’ in Japanese, which indicate ‘kidney cancer’ or ‘renal cell carcinoma’, between April 2010 and January 2020. Among the extracted patients, the inclusion criteria of this study were SBRT for tumors located in the kidney and a six-month or longer follow-up period after SBRT. A flow chart of the patient selection process is shown in Figure 1, and 29 nonconsecutive patients with 30 tumors were identified.

### Patients and tumors

The patient and tumor characteristics are shown in Table 1. One patient received a second SBRT for another tumor that developed at another site in the ipsilateral kidney 42 months after initial SBRT for kidney cancer. Three patients had previously received radical nephrectomy for kidney cancer. Four tumors were recurrent T1a (rT1a). The pathology obtained after previous surgery for kidney cancer in three patients was clear cell carcinoma, but none of the tumors from the irradiated site had histological confirmation, therefore, they were clinically diagnosed by one or more urologists and two or more radiologists using CT, MRI and ultrasonic echo findings. None of the patients received planned systemic therapy before, concomitantly or after SBRT. The median creatinine level and estimated glomerular filtration rate (eGFR) before SBRT excluding patients who had received hemodialysis (HD) before SBRT were 0.92 (interquartile range [IQR]: 0.70–1.38, range: 0.50–2.18, mg/dL) and 57.3 (IQR: 37.0–68.8, range: 24.4–114.3, ml/min/1.73 m^2^), respectively.

**Table 1:** Patient and tumor characteristics before SBRT.

Category	Variables	29 patients/30 tumors
Age, years	Median, IQR, range	71, 64–77, 49–87
Sex	Male/female	22(76%)/7(24%)
ECOG Performance Status	0/1/2/	21(72%)/7(24%)/1(3%)
Charlson comorbidity index	0/1/2/3/4–5	3(10%)/10(34%)/8(28%)/ 4(14%)/4(14%)
Collagen disease	Yes/No	2(7%)/27(93%)
	SLE/Scleroderma	1/1
Administration of antithrombotic drugs	Yes/No	14(48%)/15(52%)
History of radical nephrectomy for kidney cancer	Yes/No	3(10%)/26(90%)
Creatinine before SBRT, mg/dL	Median, IQR, range	1.18, 0.80–2.04, 0.50–8.68
Excluding HD patients (n = 27)	Median, IQR, range	0.92, 0.70–1.38, 0.50–2.18
HD before SBRT	Yes/No	5(17%)/24(83%)
Operability	Operable/Inoperable/ Undecidable	8(28%)/19(66%)/2(7%)
Reasons for choosing SBRT	Comorbidities/Renal function/Refusal of surgery	20(69%)/4(14%)/5(17%)
Implantation of fiducial markers	Yes/No	19(65%)/10(34%)
Maximum tumor diameter, mm, n = 30	Median, IQR, range	26, 22–33, 9–47
T or rT stage, n = 30	T1a/rT1a/T1b	23(77%)/4(13%)/3(10%)
N stage	N0	29 (100%)
Location of the tumor, n = 30	Right upper/mid/lower pole	6(20%)/8(27%)/2(7%)
	Left upper/mid/lower pole	5(17%)/3(10%)/6(20%)

Abbreviations: SBRT, stereotactic body radiotherapy; IQR, interquartile range; ECOG, Eastern Cooperative Oncology Group; SLE, systemic lupus erythematosus; HD, hemodialysis; rT, recurrent T.

### SBRT procedure

Our SBRT procedure and dose constraints for organs at risk were reported elsewhere [10]. Implantation of fiducial markers is typically recommended to evaluate respiratory tumor movement and to minimize internal margins. One or two fiducial markers were implanted into the renal capsule under ultrasound guidance. Twenty-one patients were implanted with fiducial markers. Each patient was immobilized in the supine position with a body frame (Vac-loc, Med-tek, Orange City, IA or VacQfix Cushion, Qfix, Avondale, PA). Respiratory tumor movement was measured using continuous X-ray images in a simulator (Ximatron or Acuity system, Varian Medical Systems, Palo Alto, CA) and/or a four-dimensional CT scan or a slow-rotation (4 sec/slice) CT scan (GE Light Speed Qxi, GE Healthcare, Waukesha, WI or SOMATOM Definition AS, Iselin, NJ). If respiratory movement of the tumor was large, an abdominal pressure belt or breath-hold technique was used. Gross tumor volume (GTV) was defined as the visible extent of the tumor on the planning CT image. If patients underwent contrast-enhanced CT, the clinical target volume (CTV) was equal to the GTV; on the other hand, the CTV was defined as the GTV plus 3 mm when patients only underwent non-contrast-enhanced CT. An internal margin was added to the CTV if needed. Finally, a planning target volume (PTV) margin of 5 mm was added to account for setup uncertainty.

The SBRT plan was created with a three-dimensional radiotherapy planning system (Eclipse, Varian Medical Systems, Palo Alto, CA). Prescription doses were based on a previous phase 1/2 study of SBRT for kidney cancer in Japan [8]. Fifty Gy in 10 fractions, 60 Gy in 10 fractions or 70 Gy in 10 fractions covering 95% of the PTV (D95) was delivered using 6–15 MV X-rays. The prescribed dose was selected based on the highest dose within dose constraints of the organ at risk (Table 2). SBRT was delivered with a linear accelerator (Clinac 23EX or TrueBeam STx, Varian Medical Systems, Palo Alto, CA) using daily image guidance.

**Table 2:** Dose constraints.

Organ at risk	Dose constraints	
Irradiated kidney (patient with two kidneys)	BED_3_ < 60 Gy	Mean
Irradiated kidney (patient with a single kidney)	BED_3_ < 50 Gy	Mean
Lung	20 Gy	≤ 20% of total volume
Spinal cord	BED_2_ < 100 Gy	Any point
Stomach and intestine	BED_3_ < 144 Gy	≤ 10 cc
	BED_3_ < 105 Gy	≤ 100 cc
Other organs	BED_3_ < 240 Gy	≤ 1 cc
	BED_3_ < 172 Gy	≤ 10 cc

Abbreviations: BED, biological effective dose, calculated by the formula BED = nd [1 + d/(α/β)], where n is the number of fractions, d is the dose/fraction, and α/β ratios are 2 Gy for the spinal cord and 3 Gy for other organs.

### Informed consent

This study was approved by the Ethics Committee of Tohoku University Hospital (reference number: 2020-1-224), and the requirement for informed consent was waived due to the retrospective study design. All patients were guaranteed the chance to opt out of participation in this study by providing them information on this study via the Internet, and opt-out consent was obtained from all patients. Furthermore, written informed consent as a part of general consent for the utilization of treatment data in future retrospective studies was obtained from all patients who were treated after April 2016.

### Definition of events

The initial tumor response was judged within six months after SBRT according to Response Evaluation Criteria in Solid Tumors (RECIST) version 1.1. DSS and OS were defined as the time from the start day of SBRT to death from kidney cancer and death from any cause, respectively. PFS was defined as the time from the start day of SBRT to any instances of recurrence or metastasis (i.e. progressive disease) or death. LC and marginal control were defined as freedom from local recurrence, which was defined as a 20% increase in the irradiated tumor volume within the PTV and recurrence in the area beyond the PTV and within the PTV plus 2 cm, respectively. LRC was defined as a combination of LC, marginal control and regional control, and regional control was defined as freedom from recurrence beyond PTV plus 2 cm and within the irradiated kidney and freedom from regional lymph node metastases (renal hilum and para-aortic region). Toxicity was judged according to the National Cancer Institute Common Terminology Criteria for Adverse Events version 5.0 translated by the Japan Clinical Oncology Group (CTCAE v5.0-JCOG).

### Statistical analyses

Time-to-event outcomes were calculated from the first day of SBRT to the day on which an event was confirmed. The confirmation of survival outcomes was performed with various methods such as telephone consultation, but other types of confirmation required some medical imaging. Cumulative incidence was calculated using the Kaplan–Meier method, and a log-rank test was used to compare Kaplan–Meier curves. Continuous covariates were divided at the sample median into two groups. A p-value less than 0.05 was defined as significant. Statistical analyses were performed using EZR version 1.52 (Saitama Medical Center, Jichi Medical University, Saitama, Japan), which is a modified version of R commander (R Foundation for Statistical Computing, Vienna, Austria) [11].

## RESULTS

SBRT was administered at a dose of 70 Gy in 10 fractions to 18 tumors, 60 Gy in 10 fractions to five tumors and 50 Gy in 10 fractions to seven tumors. The dose description of 29 tumors was D95, and only one tumor was prescribed 50 Gy to the isocenter to fulfill the dose constraints (Table 2). The median doses of the mean GTV and PTV doses were 73.8 Gy (IQR: 57.9–75.8 Gy, range: 48.5–91.8 Gy) and 72.9 Gy (IQR: 57.0–74.3 Gy, range: 47.9–80.6), respectively. The median overall treatment period was 15 days (IQR: 14–16 days, range: 12–36 days). SBRT was principally performed on consecutive days. Some patients received SBRT on nonconsecutive days because of HD, but all patients completed SBRT.

**Fig. 2.**
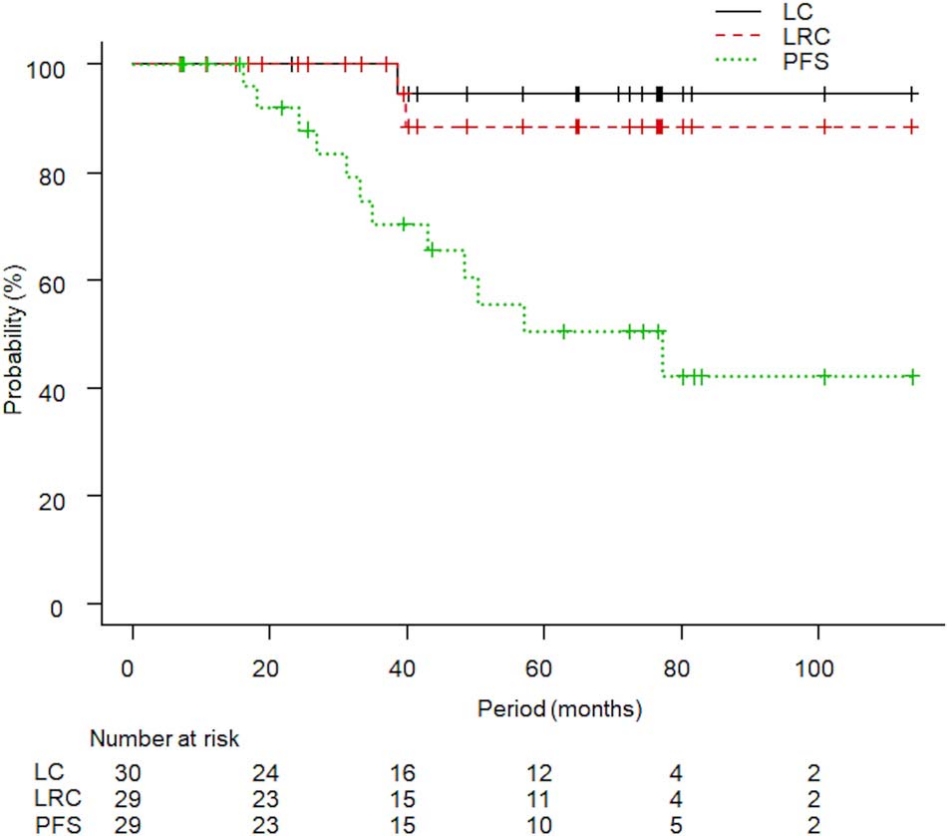
The Kaplan–Meier curves for local control (LC), locoregional control (LRC) and progression-free survival (PFS) are shown. The five-year LC, LRC and PFS rates were 94%, 88% and 50%, respectively.

**Fig. 3.**
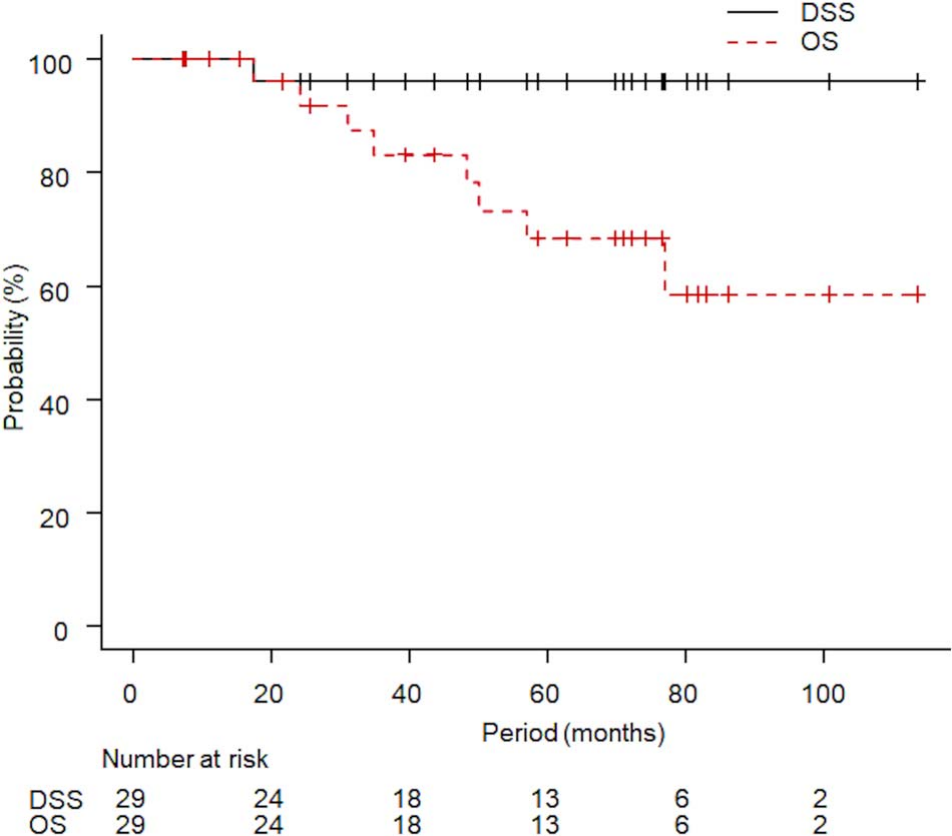
Kaplan–Meier curves for disease-specific survival (DSS) and overall survival (OS) are shown. The five-year DSS rate and OS rate were 96% and 68%, respectively.

**Fig. 4.**
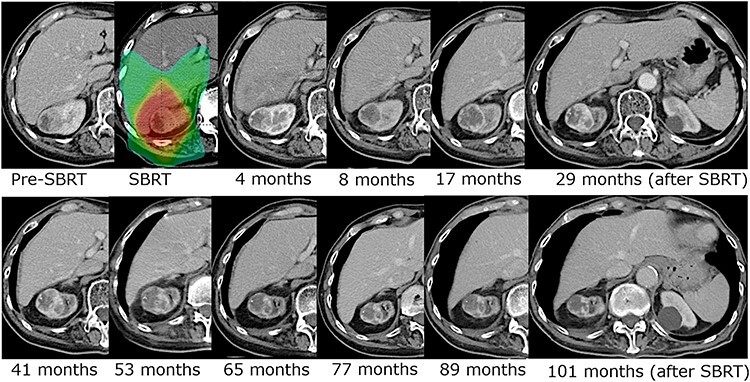
Contrast-enhanced CT images of a long-term follow-up case. Seventy Gy in 10 fractions was prescribed. The volume of the irradiated tumor and enhanced lesion increased slightly in the first four years after SBRT, and then the tumor volume decreased very slowly.

The median follow-up period for all patients was 57 months (IQR: 24.8–76.8 months, range: 7.1–113.6 months) and for survivors was 69.6 months (IQR: 23.6–81 months, range: 7.1–113.6 months). At the time of data cutoff, eight patients had died and only one patient died from kidney cancer due to metastatic disease. This patient underwent de novo SBRT for T1aN0M0 and developed multiple distant metastases (bone, liver and lung) 16 months after SBRT. Other patients died of another cancer (three patients), co-mobilities (two patients), cerebral hemorrhage (one patient) and accident (one patient). The initial tumor response after SBRT was stable disease in 22 tumors and partial response in eight tumors. Recurrence in the irradiated kidney occurred in two patients: one with local recurrence and one with recurrence outside the PTV plus 2 cm. Marginal recurrence and regional lymph node recurrence were not observed. The five-year LC rate, LRC rate and PFS rate were 94% (95% confidence interval [CI]: 66–99%), 88% (95% CI: 61–97%) and 50% (95% CI: 28–69%), respectively (Fig. 2). The five-year DSS rate and OS rate were 96% (95% CI: 61–93%) and 68% (95% CI: 44–83%), respectively (Fig. 3). A long follow-up case is shown in Fig. 4, and the irradiated tumor decreased very slowly after slight enlargement.

The patterns of the first recurrence or metastasis were as follows: bone in one patient, thyroid in one patient, lung in one patient, bone and liver in one patient and mediastinal lymph node in one patient. No regional lymph node metastasis occurred. After recurrence or metastasis, one patient received only the best supportive care. Among the other four patients, two underwent surgery, four received semi-radical irradiation and one received systemic therapy throughout the course of treatment.

Toxicities occurred in one patient with grade 1 abdominal wall pain and in one patient with grade 2 abdominal wall pain. One patient who received HD and took anticoagulants developed a chronic expanding hematoma within the irradiated volume and showed grade 1 back pain. Three of 24 non-HD patients had new-onset HD, but all of them were diagnosed with underlying kidney disease. The median peak creatinine level and eGFR within 1.5 years after SBRT in non-HD patients were 1.11 (IQR: 0.86–1.62, range: 0.48–4.58, mg/dL) and 49.3 (IQR: 32.7–62.3, range: 10.8–99.7, ml/min/1.73 m^2^), respectively. The interval between SBRT and the date when the peak value was observed was 9.9 months (IQR: 6.9–11.2, range: 2–15.7 months), and no HD was introduced during these periods. The change in the median eGFR before and after SBRT was −5.4 ml/min/1.73 m^2^ (IQR: −14.9 to −2.1, range: −43.2-22.3, ml/min/1.73 m^2^).

The results of log-rank tests for OS and PFS are shown in Table 3. No significant factor was related to OS and PFS. The smallest and the second smallest p-value for OS were operability and a history of renal surgery for kidney cancer, respectively. No operable patients (p = 0.15) or patients with a history renal surgery (p = 0.19) died.

**Table 3:** The results of log-rank tests for OS and PFS.

Category	Variables	P-value for OS	P-value for PFS
Age, years	>71 vs. ≤71	0.62	0.65
Sex	Male vs. Female	0.86	0.19
ECOG PS	0 vs. 1–2	0.37	0.08
Charlson comorbidity index	0–2 vs. 3–5	0.98	0.95
History of renal surgery for kidney cancer	Yes vs. No	0.19	0.35
HD before SBRT	Yes vs. No	0.66	0.88
Operability	Operable vs. Inoperable	0.15	0.21
Irradiated tumor diameter, mm	>26 vs. ≤26	0.32	0.58
Initial response to SBRT	SD vs. PR	0.75	0.12

Abbreviations: OS, overall survival; PFS, progression-free survival; ECOG, Eastern Cooperative Oncology Group; PS, performance status; HD, hemodialysis; SBRT, stereotactic body radiotherapy.

## DISCUSSION

The current study revealed clinical outcomes of Japanese patients treated with SBRT for kidney cancer with relatively long follow-up periods. Although OS rate was relatively low, high LC, LRC and DSS rates were achieved without grade 3 or higher toxicities. Based on these results, SBRT for kidney cancer might potentially be a treatment option for kidney cancer, but patient selection and indication for SBRT should be done carefully considering the relatively high rate of death from other causes.

The present study showed a relatively low five-year OS rate of 68% and a higher incidence of death from other causes than kidney cancer (seven deaths and one death, respectively). A report of active surveillance with delayed intervention showed a better five-year OS rate of 89% than the present study and similar tendency was seen in the report in which only five deaths in 73 were due to kidney cancer with a median follow-up period of 67 months for survivors [12]. Another report of active surveillance in elderly and/or infirm patients with T1aN0M0 kidney cancer showed only 1.1% new metastases and 12% local progression during a mean follow-up period of 28 months [13]. Local progression (recurrence) decreased when comparing the rate from the present study with the rate obtained using active surveillance. However, high LC of the present study would hardly contribute to survival considering the lower rate of OS, therefore, indications for SBRT should be carefully decided. Furthermore, when the survival outcomes of patients treated with SBRT were compared with patients receiving other treatment modalities, including partial nephrectomy and thermal ablation, SBRT resulted in significantly lower OS after propensity score matching [7]. Our survival outcome for patients treated with SBRT was almost equivalent to this previous report. SBRT for kidney cancer should not be selected as an initial treatment until favorable outcomes of SBRT have been revealed by clinical trials. Although urologists are familiar with radiotherapy through the treatment of prostate cancer, bladder cancer and other tumors, SBRT for kidney cancer should be considered only in special situations, such as tumors located near blood vessels.

LC of SBRT for kidney cancer and other types of cancer was good. The two-year estimated weighted LC rate was 92.9% in a review article of 10 publications [14]. A pooled analysis of 223 patients showed that the LC rate at both two years and four years was 97.8% [15]. The five-year LC rate of 94% in this study was consistent with these findings, and our experience suggested the importance of follow-up periods. Two cases of locoregional recurrence were documented in this study that occurred between 38 months and 40 months after SBRT (Fig. 2). The interval between SBRT and recurrence was relatively long; therefore, the difference between outcomes in short-term follow-up periods and long-term follow-up periods would be large. As a good example, a well-conceived prospective trial of The Trans-Tasman Radiation Oncology Group (TROG) 15.03 whose primary endpoint is LC requires a five-year follow-up, and the result will be reliable [16].

Figure 4 shows a long-term follow-up case with contrast-enhanced CT images. The irradiated tumor and its enhanced lesion showed a slight increase in the first four years after SBRT, and then, the tumor and enhanced lesion gradually decreased. Funayama *et al.* reported that some cases increased temporally and then showed a tendency to decrease in size [17]. The case in the present study was also supported by their findings. These phenomena might be attributed to radiation-induced changes although the results were not confirmed. Because some cases might show a delayed decrease in tumor size, some caution is needed when judging recurrence after SBRT.

In the present study, grade 3 or higher toxicities were not documented, particularly the lack of hemorrhage or perforation of the intestine. Siva *et al.* reported that the weighted rate of severe grade 3 or higher adverse events was 3.8%, ranging from 0% to 19% in the literature [14]. Kidney cancer is sometimes located near the gastrointestinal tract, of which the dose constraint might limit high-dose delivery to tumors [18]. In large-scale reports of SBRT for kidney cancer, grade 3 or higher stomach and bowel toxicities were reported, but the rate was very low (1.3%) [15]. In the present study, half of the patients took antithrombotic drugs before SBRT, and thus the safety of SBRT would be certain. From the perspective of the benefit–risk balance, the dose constraints and dose prescription strategy, which was the highest dose prescribed within the dose constraints of the organ at risk, were thought to work very well although the result of the previous phase 1/2 study have not been published [9]. Another important issue of the change in the creatinine level, particularly within 1.5 years after SBRT, has been previously reported by our institution [10]. In this subsequent follow-up study, three patients (11%) had new-onset HD, but none were judged as having SBRT-related toxicity. The mean post-SBRT change in the eGFR was reported to be −7.7 ml/min, which was similar to the value in the current study (−6.1 ml/min/1.73 m^2^); therefore, SBRT was safe and is recommended for patients with one functioning kidney [6,19].

The present study has some limitations. This study was a retrospective single-institute study. The number of patients was small; therefore, multivariate analyses were unable to be performed. Furthermore, the benefit of SBRT for T1 or rT1 kidney cancer has not been reported. Some confounding factors exist because of the retrospective nature of the study. One patient with a very short follow-up period (≤6 months) was excluded from this study and some patients with a short follow-up period were included in the study, which would affect the results.

In conclusion, SBRT for kidney cancer showed high LC, LRC and DSS rates. The low rate of toxicity and lack of severe toxicity in this study confirmed the safety of SBRT in Japanese patients. But the OS rate was not good, therefore patient selection and indication for SBRT should be done carefully.

## ABBREVIATIONS

CT – computed tomography; MRI – magnetic resonance imaging; SBRT – stereotactic body radiotherapy; NCCN – National Comprehensive Cancer Network; OS – overall survival; LC – local control; LRC – locoregional control; PFS – progression-free survival; DSS – disease-specific survival; eGFR – estimated glomerular filtration rate; HD – hemodialysis; IQR – interquartile range; GTV – gross tumor volume; CTV – clinical target volume; PTV – planning target volume; D95 – covering 95% of the planning target volume; RECIST – Response Evaluation Criteria in Solid Tumors; CTCAE v5.0-JCOG – the National Cancer Institute Common Terminology Criteria for Adverse Events version 5.0 translated by the Japan Clinical Oncology Group; CI – confidence interval.
